# Management of Caesarean scar pregnancy with or without methotrexate before curettage: human chorionic gonadotropin trends and patient outcomes

**DOI:** 10.1186/s12884-018-1923-x

**Published:** 2018-07-04

**Authors:** Sheng Wang, Rajluxmee Beejadhursing, Xiangyi Ma, Ya Li

**Affiliations:** Department of Obstetrics and Gynecology, Tongji Hospital, Tongji Medical College, Huazhong University of Science and Technology, Wuhan, 430030 People’s Republic of China

**Keywords:** Cesarean scar pregnancy, Human chorionic gonadotropin, Methotrexate, Suction curettage, Protocol comparisons

## Abstract

**Background:**

To evaluate the effects of systemic methotrexate in cesarean scar pregnancy (CSP) patients treated with ultrasound-guided suction curettage.

**Methods:**

A retrospective review of all women presenting with CSP treated with ultrasound-guided suction curettage at Tongji Hospital, Wuhan, China, between January 1, 2013 and December 31, 2015, was conducted. Patients were grouped into those not treated with methotrexate before curettage (group 1), treated with methotrexate by intramuscular injection (group 2) and treated with methotrexate by intravenous injection (group 3). The clinical characteristics and outcomes were analyzed.

**Results:**

Among 107 patients, 47 patients were not treated with methotrexate before curettage, 46 patients had methotrexate administered by intramuscular injection and 14 patients had methotrexate injected intravenously. There were no significant differences among the groups in basic and clinical characteristics, such as age, gravity, parity, positive fetal heart beat and gestational age at diagnosis. Patients presented similar initial human chorionic gonadotropin (hCG) levels in all groups. After treatment with methotrexate or curettage, the percentage changes and varied ranges of the hCG levels were also similar in all groups. There were no significant differences in intraoperative blood loss and retained products of conception among the three groups. However group 1 had significantly shorter hospital stays than the two groups that were treated with methotrexate (*p*<0.001).

**Conclusion:**

By grouping CSP patients who shared similar age, gravity, parity, fetal heart beat positive and gestational age at diagnosis, we found that the presence or absence of methotrexate treatment before curettage resulted in comparable outcomes and hCG levels, although patients who were not treated with methotrexate had significantly shorter stays in the hospital.

## Background

Cesarean scar pregnancy (CSP) is defined as the implantation of a gestational sac within the scar of a previous cesarean surgery. If CSP is maintained, there are potentially higher risks that include uterine rupture, devastating hemorrhage, loss of subsequent fertility and even maternal mortality [1]. Therefore, the standard protocol for CSP management is to terminate the pregnancy. Nevertheless, while the optimal management of CSP remains unclear, there is a variety of therapeutic strategies are currently in use, such as medical treatment with systemic MTX, medical treatment with systemic and local MTX, uterine curettage, hysteroscopy, resection of CSP through a transvaginal approach, uterine artery embolization, laparoscopy, and high-intensity focused ultrasound [2]. Some complicated case may require combined application of several methods [3].

MTX is a folic acid antagonist that inhibits the enzyme dihydrofolate reductase, thereby interfering with DNA synthesis in rapidly dividing cells such as trophoblasts. The first usage of MTX in the treatment of ectopic pregnancy was reported by Tanaka et al. in an interstitial ectopic pregnancy in 1982 [4]. Recently, MTX has also used in treatment of cervical pregnancy, which is another form of ectopic pregnancy [5]. CSP is a special type ectopic pregnancy and as such, some researchers have investigated the use of MTX in conservative management or preliminary treatment of CSP prior to curettage [6, 7]. The application of the systemic administration of MTX in CSP is controversial. Semih Z. reported that systemic MTX manifested similar results compared to local MTX in terms of treatment success and reproductive outcomes [8]. Some researchers have further suggested that systemic MTX may be less effective in cases with higher serum hCG concentrations [9]. The effects of systemic MTX before curettage and whether it is recommended before curettage are still unknown. The aim of this study was to evaluate the hCG levels and outcomes in CSP patients treated with or without MTX before curettage to assess the efficacy of MTX.

## Methods

This retrospective study analysed CSP patients treated at the Tongji Hospital, Wuhan, China, between 2013 and 2015. Data were collected through archived medical records. The study was approved by the hospital’s ethics committee. Due to the retrospective nature of this study, informed consent from the patients was not deemed necessary at the time of data collection. However, the authors wish to disclose that, in accordance with hospital policy, patients were clearly informed of their treatment modalities and provided signed consent prior to their intervention. A diagnosis of CSP relied on routine sonographic criteria, including 1) empty uterine cavity and cervical canal; 2) a gestational sac located anteriorly at the level corresponding to the prior lower uterine segment of the cesarean section scar; 3) evidence of functional trophoblastic/placental circulation on Doppler scans; and 4) a negative sliding organs sign, which was defined as the inability to displace the gestational sac from its position at the level of the internal os [10]. Patients who were unsuccessfully treated and then transferred to our hospital were excluded from this study.

The protocol for treating CSP in our hospital included suction evacuation, chemotherapy with MTX, hysteroscopy, laparoscopy, transabdominal approach or a combination of two or more treatment options. In this study, our aim was to only assess the effects of MTX used before curettage. In the assessment of treatment outcome, we included patients who met the following criteria: (1) they were diagnosed with CSP and (2) either underwent medical treatment with MTX by intramuscular injection, by intravenous injection or were not treated with MTX before ultrasound-guided suction curettage. MTX was administered according to the protocol originally described by Stovall et al. [11] in 1991. In brief, MTX was administered intramuscularly two times (Day 0 and Day 4) or intravenously for 5 days (Day 0 to Day 5), with the same total dose of 50 mg/m^2^. Suction curettage was performed the day following MTX treatment without awaiting a decline in serum hCG levels.

The clinical characteristics of the patients, such as age, gravity, parity, and number of previous cesarean sections, were reviewed. Retained products of conception, intraoperative blood loss and duration of hospital were analyzed. The volume of blood lost during surgery was determined by measuring contents of the suction container. In all patients, any material aspirated from the uterus was collected in suction container. After operation, the container was filtered with a screen filter, and the blood was measured in a graduated volume flask. Any other significant blood loss collected on surgical swabs and drapes and was subjectively assessed by the operating surgeon. Three days after operation, a transvaginal scan was performed to verify any retained products of conception, and serum hCG was also performed. If the scans were cleared and serum hCG had markedly declined by more than half of the pre-operation value, patients were subsequently discharged. Statistical analysis was performed using the IBM SPSS version 17.0 software (SPSS Inc., Chicago, IL). Quantitative data are presented as the mean ± SD, frequency and percentage; between-group differences were assessed by Student’s *t*-test for continuous variables and the chi-squared test for categorical variables. *P*-values < 0.05 were considered indicative of a statistically significant difference.

## Results

During the study period, our institution received a total of 317 patients with CSP; 107 patients underwent ultrasound-guided suction curettage, of which 47 patients were not treated with MTX before curettage, 46 patients were treated with MTX intramuscularly and 14 patients were treated with MTX by intravenous injection before curettage.

The baseline characteristics of the patients in the 3 groups are described in Table 1. The incidence of previous cesarean sections was significantly different among the 3 groups (1.17 ± 0.380, 1.17 ± 0.383, and 1.57 ± 0.514, respectively; *P* = 0.004). There were no significant differences between the 3 groups with respect to age, gravity, parity, fetal heart beat positive and gestational age at diagnosis.

**Table 1:** Comparison of the basic and clinical characteristics of patients not treated with MTX (Group 1), treated with MTX by intramuscular injection (Group 2) or treated with MTX by intravenous injection (Group 3)

	Mean ± SD/Rate			*P* value
	Group 1(*n* = 47)	Group 2(*n* = 46)	Group 3(*n* = 14)	
Age (years)	32.04 ± 6.283	31.15 ± 5.597	34.14 ± 4.865	0.206
Previous cesarean section (n)	1.17 ± 0.380	1.17 ± 0.383	1.57 ± 0.514	0.004
Gravidity (n)	4.13 ± 1.24	4.20 ± 1.470	5.0 ± 1.519	0.186
Parity (n)	1.21 ± 0.414	1.22 ± 0.513	1.50 ± 0.519	0.08
Gestational age at diagnosis (days)	52.68 ± 11.66	48.76 ± 8.945	52.15 ± 13.619	0.292
Fetal heart beat positive, n (%)	15(31.9%)	21(45.7%)	5(35.7%)	0.466

Patients had similar initial hCG levels before MTX administration in group 2 and group 3 (Table 2). When we examined hCG trends, we found that the mean decline of hCG after MTX treatment was not significantly different between group 2 and group 3 (*p* = 0.196). In both surveys before and 3 days after curettage, the average hCG levels of the 3 groups were similar. We further examined the range of the changes in hCG levels and observed that after MTX treatment, the varied ranges and percentage changes of hCG levels were similar in group 2 and group3, as shown in Table 2 B-A and (B-A)/A (*p* = 0.219 and *p* = 0.273, respectively). Three days after curettage, the hCG percentage changes were also similar among the 3 groups as shown in Table 2 C/B (*p* = 0.674).

**Table 2:** Human chorionic gonadotropin levels (mIU/ml) in patients not treated with MTX (Group 1), treated with MTX by intramuscular injection (Group 2) or treated with MTX by intravenous injection (Group 3)

	Mean ± SD/Rate			*P* value
	Group 1(*n* = 47)	Group 2(*n* = 46)	Group 3(*n* = 14)	
A:Before MTX treatment	–	44,603.28 ± 38,615.27	25,648.86 ± 16,125.92	0.080
B:After MTX treatment or before curettage	31,163.48 ± 28,626.86	37,712.91 ± 23,880.85	31,005.21 ± 23,029.37	0.196
C:Three days after curettage	2838.68 ± 3.10	4434.98 ± 4.90	3263.46 ± 2.93	0.077
B-A	–	9664.21 ± 8993.10	22,506.29 ± 36,916.63	0.219
(B-A)/A	–	0.27 ± 0.213	1.33 ± 3.451	0.273
C/B	0.085 ± 0.04	0.084 ± 0.05	0.79 ± 0.04	0.674

The outcomes of the treatment are shown in Table 3. There were no significant differences in intraoperative blood loss and rates of retained products of conception among the 3 groups (*p* = 0.066 and *p* = 0.658, respectively). Duration of hospitalisation was significantly shorter in group 1 (*p*<0.001).

**Table 3:** Comparison of outcomes for patients not treated with MTX (Group 1), treated with MTX by intramuscular injection (Group 2) or treated with MTX by intravenous injection (Group 3)

	Mean ± SD/Rate			*P* value
	Group 1(*n* = 47)	Group 2(*n* = 46)	Group 3(*n* = 14)	
Intraoperative blood loss (ml)	39.83 ± 16.96	55.33 ± 44.19	42.86 ± 22.68	0.066
Pregnancy tissue retained, n (%)	1(2.1%)	2(4.3%)	1(7.1%)	0.658
Hospital stay (days)	7.00 ± 2.29	9.59 ± 2.46	13.86 ± 3.88	<0.001

Data comparing patients who underwent MTX therapy with patients who did not is displayed in Table 4. The outcomes of the treatment are the same when we further divided the MTX group according to mode of administration; by intramuscular injection or by intravenous injection. There were no significant differences in intraoperative blood loss and rates of retained products of conception among MTX patients and patients who were not treated with MTX (*p* = 0.051 and *p* = 0.437, respectively). Duration of hospital stay was significantly shorter in the group that did not receive MTX (*p*<0.001).

**Table 4:** Comparison basic clinical characteristics and outcomes of patient not treated by MTX or treated by MTX

	Mean ± SD/Rate		*P* value
	Not treated by MTX (*n* = 47)	treated by MTX (*n* = 60)	
Age (years)	32.04 ± 6.283	31.85 ± 5.544	0.867
Gestational age at diagnosis (days)	52.68 ± 11.664	49.37 ± 10.096	0.119
Fetal heart beat positive, n (%)	15(31.9%)	26(43.3%)	0.299
B:After MTX treatment or before curettage (mIU/ml)	31,163.48 ± 28,626.86	52,841.27 ± 41,148.83	0.003
C:Three days after curettage (mIU/ml)	2838.68 ± 3.10	4161.63 ± 4.52	0.090
C/B	0.085 ± 0.04	0.082 ± 0.05	0.856
Intraoperative blood loss (ml)	39.83 ± 16.96	52.37 ± 40.2	0.051
Pregnancy tissue retained, n (%)	1(2.1%)	3(5.0%)	0.437
Hospital stay (days)	7.00 ± 2.29	10.58 ± 3.35	<0.001

## Discussion

In this study, the efficacy of MTX use before curettage in managing CSP was assessed. The clinical outcomes of the three groups (groups 1 to 3) assessed in this study were similar, with intraoperative blood loss of 39.83 ± 16.96 ml, 55.33 ± 44.19 ml and 42.86 ± 22.68 ml, respectively. Assuming the lack of retained products of conception as a clinical success, we observed success rates of 97.9, 95.7 and 92.9%, respectively. We also found that using MTX before curettage, either by intramuscular or intravenous injection, could significantly prolong the hospital-stay duration of patients.

CSP can be classified into several subtypes based on different conditions, such as the degree of invasion of the amniotic sac, gestational age at diagnosis, positive fetal heart beat and myometrial thickness of the lower segment. The optimal management of CSP is unclear and a variety of therapeutic strategies are being used, with presently no clearly defined protocol consensus. Özkan Özdamar et al. demonstrated that in appropriate CSP cases, ultrasound-guided suction curettage appears to be a reliable treatment option. [12]. Jurkovic D. also considered suction curettage as an effective method for the treatment of CSP which was associated with a lower risk of blood transfusion and hysterectomy [13]. Nonetheless, several studies have reported that suction curettage should not be considered as an optimal first line of therapy [9, 14]. Different patients with a wide range of conditions who visit diverse doctors are definitely treated differently. The same situation occurs in our hospital. In the present study, we analyzed 107 patients with similar basic and clinical characteristics who underwent ultrasound-guided suction curettage. Before curettage, some patients were treated with MTX, either by intramuscular or intravenous injection.

Systemic and local MTX are two of the most widely used forms of management for CSP. Previous studies comparing the two treatments showed conflicting results likely due to differences in study design, supplementary treatments and various definitions of response [15, 16]. A recent review [2] showed that almost every fourth woman treated with systemic MTX required additional treatment, and severe complications occurred in 13% of them. Therefore, one may wonder whether chemotherapy using MTX is truly justified. Our present study showed similar outcomes among the three groups studied. Even though patients underoging MTX treatment, had elevated hCG levels after the initial dose, 3 days after curettage, the hCG percentage change was also the same whether patients were treated with MTX or not. In theory, MTX may take some time to cause trophoblastic cytolysis. In addition, MTX half-life is short, making it difficult for MTX to reach the sac and embryo [17]. Owing to of these setbacks, trophoblastic cells in CSP may be insensitive to MTX. Another possibility is that not enough time was given after the MTX treatment to observe a responsive effect. In consideration of these possibilities that may confound the effects of MTX, we further analyzed the outcomes of the 3 groups.

Massive hemorrhage is a major complication of CSP. In this study, the means of intraoperative blood loss were similar in all three groups. Only 1 patient in group 2 reported a blood loss of more than 300 ml and subsequently underwent a laparoscopy. None of the patients suffered from uterine rupture and devastating hemorrhage. Perhaps, our results differed from other reports, because of differences in the selection criteria used when determining which patients could be treated with suction curettage. Retention of products of conception after CSP treatment is a major concern for both patients and their physicians, as it may adversely influence menstruation and any future fertility wishes. In this present study, among the 3 groups, respectively 1 (2.1%), 2 (4.3%), and 1 (7.1%) patients, reported retained products of conception and required further treatment. These findings revealed no significant differences between the 3 groups. Our hospital records have no instances where only MTX was used to treat CSP and as a result, we could not assess the outcomes of standalone MTX treatment. Hua Yang [18] reported that conservative MTX treatment achieved satisfactory therapeutic effects, similar to uterine artery embolization (UAE) group, but the recovery time was significantly longer than that of the curettage group. In our study, the duration of hospitalization was also significantly longer in the MTX groups. Therefore, based on our results, MTX administration before curettage has no obvious remedial effects in patients managed with ultrasound-guided suction curettage and has an additional side effect of prolonging hospital-stay.

This studying is primarily limited by its retrospective nature. However, it would have been ethically inappropriate to compare our results with a control group because some of these procedures would need to be thouroughly explained before approval of patients which would induce various sorts of bias and interference originating from the authors, hospital personnel or patients. Another limitation we wish to disclose is that subsequent fertility outcomes of the different treatment groups was unfeasible in the present setting and hence not investigated.

## Conclusions

In conclusion, all the CSP patients treated with ultrasound-guided suction curettage reported satisfactory results. Besides, the use of MTX before curettage had no significant effects on intraoperative blood loss or retention of pregnancy tissue. Concurrently, the duration of hospital stay of patients treated with MTX was significantly prolonged.

## Abbreviations

CSP: Cesarean scar pregnancy
hCG: human chorionic gonadotropin
MTX: methotrexate
UAE: uterine artery embolization

## References

[1] Rotas MA, Haberman S, Levgur M (2006). Cesarean scar ectopic pregnancies: etiology, diagnosis, and management. Obstet Gynecol.

[2] Birch PK, Hoffmann E, Rifbjerg LC, Svarre NH (2016). Cesarean scar pregnancy: a systematic review of treatment studies. Fertil Steril.

[3] Giampaolino P, Della Corte L, Venetucci P, D'Antuono F, Morra I, Nappi C, et al. Treatment of asymptomatic uterine rupture of caesarean scar pregnancy in patient with advanced gestational age: case report. J Obstet Gynaecol. 2017:1–2. 10.1080/01443615.2017.1363171 29057686

[4] Tanaka T, Hayashi H, Kutsuzawa T, Fujimoto S, Ichinoe K (1982). Treatment of interstitial ectopic pregnancy with MTX: report of a successful case. Fertil Steril.

[5] Di Spiezio Sardo A, Vieira MDC, Laganà AS, Chiofalo B, Vitale SG, Scala M (2017). Combined systemic and Hysteroscopic intra-amniotic injection of methotrexate associated with Hysteroscopic resection for cervical pregnancy: a cutting-edge approach for an uncommon condition. Eurasian J Med.

[6] Haimov-Kochman R, Sciaky-Tamir Y, Yanai N, Yagel S (2002). Conservative management of two ectopic pregnancies implanted in previous uterine scars. Ultrasound Obstet Gynecol.

[7] Litwicka K, Greco E (2011). Caesarean scar pregnancy: a review of management options. Curr Opin Obstet Gynecol.

[8] Uludag SZ, Kutuk MS, Ak M, Ozgun MT, Dolanbay M, Aygen EM (2016). Comparison of systemic and local MTX treatments in cesarean scar pregnancies: time to change conventional treatment and follow-up protocols. Eur J Obstet Gynecol Reprod Biol.

[9] Seow KM, Huang LW, Lin YH, Lin MY, Tsai YL, Hwang JL (2004). Cesarean scar pregnancy: issues in management. Ultrasound Obstet Gynecol.

[10] Jurkovic D, Hillaby K, Woelfer B, Lawrence A, Salim R, Elson CJ (2003). First-trimester diagnosis and management of pregnancies implanted into the lower uterine segment cesarean section scar. Ultrasound Obstet Gynecol.

[11] Stovall TG, Ling FW, Gray LA (1991). Single-dose MTX for treatment of ectopic pregnancy. Obstet Gynecol.

[12] Özdamar Ö, Doğer E, Arlıer S, Çakıroğlu Y, Ergin RN, Köpük ŞY (2016). Exogenous cesarean scar pregnancies managed by suction curettage alone or in combination with other therapeutic procedures: a series of 33 cases and analysis of complication profile. J Obstet Gynaecol Res.

[13] Jurkovic D, Knez J, Appiah A, Farahani L, Mavrelos D, Ross JA (2016). Surgical treatment of cesarean scar ectopic pregnancy: efficacy and safety of ultrasound-guided suction curettage. Ultrasound Obstet Gynecol.

[14] Ayoubi JM, Fanchin R, Meddoun M, Fernandez H, Pons JC (2001). Conservative treatment of complicated cesarean scar pregnancy. Acta Obstet Gynecol Scand.

[15] Peng P, Gui T, Liu X, Chen W, Liu Z (2015). Comparative efficacy and safety of local and systemic MTX injection in cesarean scar pregnancy. Ther Clin Risk Manag.

[16] Cok T, Kalayci H, Ozdemir H, Haydardedeoglu B, Parlakgumus AH, Tarim E (2015). Transvaginal ultrasound-guided local MTX administration as the first-line treatment for cesarean scar pregnancy: follow-up of 18 cases. J Obstet Gynaecol Res.

[17] Kutuk MS, Uysal G, Dolanbay M, Ozgun MT (2014). Successful medical treatment of cesarean scar ectopic pregnancies with systemic multidose MTX: single-center experience. J Obstet Gynaecol Res.

[18] Yang H, Li S, Ma Z, Jia Y (2016). Therapeutic effects of uterine artery embolisation (UAE) and MTX (MTX) conservative therapy used in treatment of cesarean scar pregnancy. Arch Gynecol Obstet.

